# Geminin Escapes Degradation in G1 of Mouse Pluripotent Cells and Mediates the Expression of Oct4, Sox2, and Nanog

**DOI:** 10.1016/j.cub.2011.03.026

**Published:** 2011-04-26

**Authors:** Valerie S. Yang, Stephanie A. Carter, Sarah J. Hyland, Kikuë Tachibana-Konwalski, Ronald A. Laskey, Michael A. Gonzalez

**Affiliations:** 1 MRC Cancer Cell Unit, Hutchison/MRC Research Centre, Hills Road, Cambridge CB2 0XZ, UK; 2 Department of Biochemistry, University of Oxford, South Parks Road, Oxford OX1 3QU, UK

## Abstract

Geminin is an essential cell-cycle protein that is only present from S phase to early mitosis in metazoan somatic cells [1, 2]. Genetic ablation of geminin in the mouse results in preimplantation embryonic lethality because pluripotent cells fail to form and all cells differentiate to trophoblast [3, 4]. Here we show that geminin is present in G1 phase of mouse pluripotent cells in contrast to somatic cells, where anaphase-promoting complex/cyclosome (APC/C)-mediated proteasomal destruction removes geminin in G1 [1, 2, 5]. Silencing geminin directly or by depleting the APC/C inhibitor Emi1 causes loss of stem cell identity and trophoblast differentiation of mouse embryonal carcinoma and embryonic stem cells. Depletion of cyclins A2 or B1 does not induce this effect, even though both of these APC/C substrates are also present during G1 of pluripotent cells. Crucially, geminin antagonizes the chromatin-remodeling protein Brg1 to maintain expression of Oct4, Sox2, and Nanog. Our results define a pluripotency pathway by which suppressed APC/C activity protects geminin from degradation in G1, allowing sustained expression of core pluripotency factors. Collectively, these findings link the cell cycle to the pluripotent state but also raise an unexplained paradox: How is cell-cycle progression possible in pluripotent cells when oscillations of key regulatory proteins are lost?

## Highlights

► Geminin is essential for pluripotency of mouse EC and ES cells ► Accordingly, mouse EC and ES cells retain geminin in G1 ► By activating APC/C^Cdh1^, Emi1 depletion phenocopies depletion of geminin ► Geminin sustains core pluripotency factors by antagonizing chromatin remodeler Brg1

## Results and Discussion

The generation of induced pluripotent stem (iPS) cells is an important but highly inefficient process [6]. A major barrier that is likely to attenuate reprogramming efficiency is the cell cycle, which must be modified in mammalian somatic cells to one that precisely mirrors the cell cycle of embryonic stem (ES) cells. In contrast to somatic cells, G1 phase of the cell cycle is severely truncated in ES cells [7]. In mouse ES cells, cyclin-dependent kinase (cdk) activity is unopposed [8, 9] and the restriction point is compromised because of a constitutively hyperphosphorylated retinoblastoma protein [10]. Together, these important differences indicate that intrinsic regulation of the cell cycle might be critical in sustaining the pluripotent state. Accordingly, c-Myc expression [11, 12], repression of the *INK4/Arf* locus [13], and inactivation of the tumor suppressor p53 [14] are all strategies that perturb the somatic cell cycle and enhance the efficiency of nuclear reprogramming in the generation of iPS cells.

We set out to investigate the requirement of crucial cell-cycle regulators in maintaining the identity and genome ploidy of pluripotent cells by transient transfection of small interfering RNA (siRNA) oligonucleotides using both mouse embryonal carcinoma (EC) and ES cells. We focused initially on geminin because we previously observed that mouse embryos that are null for geminin not only are preimplantation lethal [3, 4] but also fail to form pluripotent cells [3]. Instead, they form only trophoblast giant cells. Geminin is a cell-cycle regulator in multicellular eukaryotes that inhibits prereplication complex assembly between S phase and the metaphase-anaphase transition by preventing Cdt1 from recruiting minichromosome maintenance proteins to chromatin [1–3]. Geminin also couples cell-cycle control with differentiation during neural development by interacting with Brg1 [15], Six3 [16], Hox, and Polycomb complex proteins [17].

**Figure 1:**
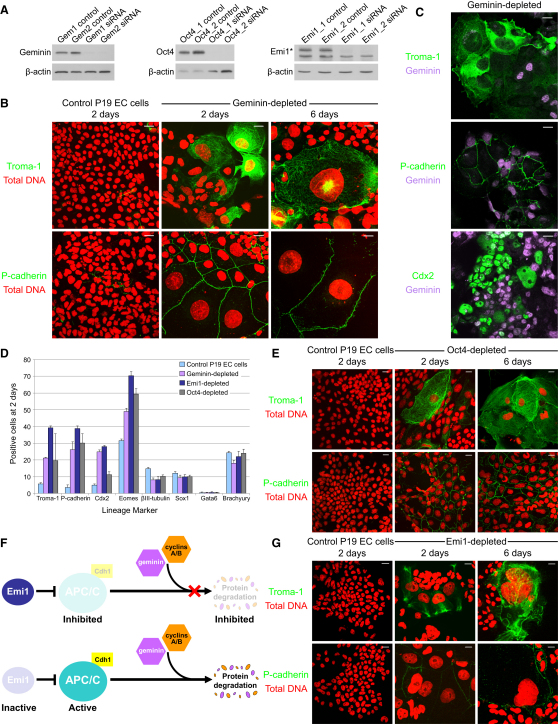
Silencing Geminin or Emi1 Mimics Depletion of the Pluripotency Factor Oct4 in P19 Mouse Embryonal Carcinoma Cells (A) Geminin, Oct4, and Emi1 were depleted by siRNA in P19 embryonal carcinoma (EC) cells, and whole-cell lysates were harvested for analysis by western blotting. β-actin was used as a loading control. In mouse cells, a cross-reacting band lies below the Emi1 band marked with an asterisk. (B) Geminin depletion by siRNA in P19 EC cells results in upregulation of trophectoderm markers Troma-1 and P-cadherin (both in green) and nuclear enlargement at 2 days and 6 days posttransfection. Total DNA is shown in red. Scale bars represent 20 μm. (C) Trophectoderm markers Troma-1, P-cadherin, and Cdx2 (in green) are induced in P19 EC cells that lack geminin (purple). The mean proportion of cells lacking geminin was 67.8% of cells at 24 hr versus 43% at 48 hr as a result of outgrowth of untransfected cells (data not shown). (D) Bar chart showing lineage analysis of control, geminin-, Oct4-, and Emi1-depleted P19 EC cells. The proportion of cells labeled by immunofluorescence with markers of extraembryonic (Troma-1, P-cadherin, Cdx2) and embryonic (β-III tubulin, Sox1, Gata6, and Brachyury) differentiation is shown. Eomes is a marker of both mesoderm and trophoblast differentiation. At least 1000 cells were scored, and all cells in a given field were scored. Error bars represent 5% standard error. (E) Upregulation of trophectoderm markers is observed in P19 EC cells depleted of Oct4 at 2 days and 6 days posttransfection. (F) Emi1 inhibits APC/C^Cdh1^ activity to drive progression through the mitotic cell cycle by inhibiting the degradation of geminin and cyclins A2 and B1. Emi1 inactivation (or depletion) results in unopposed APC/C^Cdh1^ activity and ubiquitin-mediated proteasomal destruction of its substrates geminin and cyclins. (G) Emi1 depletion phenocopies depletion of geminin in P19 EC cells with upregulation of trophectoderm markers and gross nuclear enlargement observed at 2 days and 6 days. Total DNA is shown in red. Scale bars represent 20 μm. For additional related data, see Figure S1.

We first depleted geminin from asynchronous mouse P19 EC cells (Figure 1A), which are capable of embryonic and extraembryonic differentiation [18]. This resulted in massive nuclear enlargement (Figure 1B). Nuclear size was greater at 6 days than at 2 days posttransfection, and the extent of nuclear enlargement was much greater in EC cells than in mouse 3T3 fibroblasts (data not shown). Strikingly, depletion of geminin in P19 EC cells mimics depletion of Oct4 [18] (also known as Pou5f1; Figures 1A–1E), a core transcription factor required for self-renewal in ES cells [19]. Depletion of geminin in P19 EC cells induces markers of trophoblast differentiation (Figure 1B). Thus, immunofluorescent staining with Troma-1, a trophectoderm-specific monoclonal antibody raised against cytokeratin endo-A [20], showed upregulation in geminin-depleted cells (Figures 1C and 1D). Placental cadherin (P-cadherin, cadherin-3, Cdh3), the caudal-type homeodomain transcription factor Cdx2, and eomesodermin (Eomes) were also upregulated in geminin-depleted P19 EC cells, but transcription factors associated with other developmental lineages were not (Figure 1D; see also Figure S1 available online). We conclude that geminin depletion causes extraembryonic differentiation into trophoblast, and not differentiation into other lineages.

Geminin levels in wild-type trophoblast giant cells are much lower than in their pluripotent counterparts as a result of proteasomal destruction [3], suggesting that loss of geminin from trophoblast cells might be mediated by extended activity of the anaphase-promoting complex/cyclosome (APC/C) during interphase. In association with specific coactivators, the multisubunit APC/C polyubiquitinates cell-cycle regulatory proteins in somatic cells, such as cyclins and Cdk inhibitors, targeting them for destruction by the 26S proteasome during mitosis and G1 [21] (Figure 1F). Two coactivator proteins of the APC/C are Cdc20 and Cdh1. APC/C^Cdc20^ is active from prophase to the metaphase-anaphase transition. At the metaphase-anaphase transition, APC/C^Cdh1^ activity increases and persists throughout the following G1 until the onset of DNA replication, when inhibition by early mitotic inhibitor 1 (Emi1) during S phase prevents further APC/C^Cdh1^ activity until prophase [22, 23]. To investigate the role of APC/C^Cdh1^ in pluripotent cells, we depleted its negative regulator, Emi1. Figure 1G shows that in P19 EC cells, depletion of Emi1 also causes giant cell formation and upregulation of the trophectoderm markers Troma-1, P-cadherin, Cdx2, and Eomes (Figure 1G; Figure S1). To verify that APC/C^Cdh1^ is responsible for inducing the observed phenotype in pluripotent cells depleted of Emi1, cells were cotransfected with siRNA duplexes targeting Cdh1, Emi1, or both together. Codepleting Emi1 and Cdh1 antagonizes the effect of depleting Emi1 alone, because nuclear size at 2 days posttransfection was comparable to control cells and considerably smaller than that of Emi1-depleted cells (Figure S2A). Moreover, codepleting Cdh1 also inhibited upregulation of trophectoderm markers, indicating that giant cell formation and trophoblast differentiation in pluripotent cells depleted of Emi1 requires APC/C^Cdh1^.

**Figure 2:**
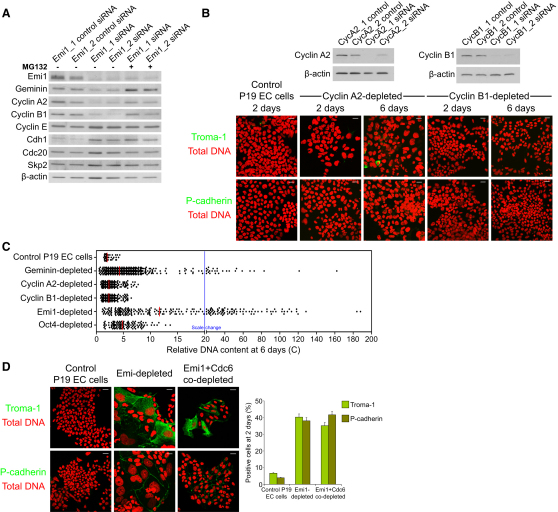
Emi1 Depletion Induces Trophoblast Differentiation in Mouse EC Cells by Downregulation of Geminin, but Not by Downregulation of Cyclins A2 or B1 (A) Emi1 depletion in P19 EC cells resulted in downregulation of geminin and cyclins A2 and B1 by immunoblotting, with stabilization of each of these APC/C substrates following treatment with the proteasome inhibitor MG132. (B) P19 EC cells were transfected with cyclin A2 or cyclin B1 siRNA, and whole-cell lysates were harvested for analysis by western blotting. β-actin was used as a loading control. Giant cell formation and upregulation of Troma-1 and P-cadherin are not evident following depletion of cyclin A2 or cyclin B1. Total DNA is shown in red. Scale bars represent 20 μm. (C) Scatter dot plots show relative DNA content in geminin-, cyclin A2-, cyclin B1-, Emi1-, and Oct4-depleted P19 EC cells relative to control siRNA-treated cells. (D) Codepletion of Emi1 and Cdc6 abolishes nuclear enlargement, but not upregulation of trophectoderm markers Troma-1 and P-cadherin (both green), in P19 EC cells at 2 days posttransfection. Total DNA is shown in red. Scale bars represent 20 μm. Bar chart shows the proportion of control, Emi1-depleted, and Emi1- and Cdc6-codepleted P19 EC cells labeled with Troma-1 and P-cadherin by immunofluorescence. At least 1000 cells were scored for each time point. Error bars represent 5% standard error. For additional related data, see Figure S2.

We next examined whether APC/C^Cdh1^ substrates were destabilized following depletion of Emi1 in P19 EC cells, particularly because geminin, cyclin A2, and cyclin B1 are crucial for cell-cycle regulation and maintenance of a diploid DNA content in somatic cells. Geminin and cyclin A2-Cdk2 (and Cdk1) prevent illegitimate assembly of prereplication complexes on chromatin when APC/C activity is low in somatic cells during S phase and G2 [1, 2, 24, 25], whereas cyclin B1-Cdk1 initiates mitosis and is required for its progression [26]. We found that Emi1 depletion resulted in significant downregulation of geminin, cyclin A2, and cyclin B1 (Figure 2A; Figures S2B and S2C), as previously observed in somatic cells [22, 23]. Cyclin E, which is targeted for proteasomal degradation by the SCF^Skp2^ ubiquitin ligase [27] and not the APC/C, was upregulated by immunofluorescence in enlarged nuclei depleted of Emi1 (Figure S2D). Geminin, cyclin A2, and cyclin B1 were downregulated in pluripotent cells depleted of Emi1 and dramatically stabilized following treatment with the proteasome inhibitor MG132 (Figure 2A; Figure S2D). When we depleted P19 EC cells of cyclin A2, however, cyclin A2-depleted cells showed minimal upregulation of trophectoderm markers, with only a modest increase in DNA content to 4C that could be attributed to a G2 arrest (Figures 2B and 2C), as observed in somatic cells that have been depleted of cyclin A2 [28]. Cyclin B1 is also a target of APC/C^Cdh1^, and although committed trophoblast stem cells are able to endoreduplicate following p57^Kip2^-mediated inhibition of Cdk1 [29], we found that depletion of cyclin B1 alone in pluripotent cells failed to result in nuclear enlargement or upregulation of trophectoderm markers (Figure 2B). These results show that both trophoblast differentiation and giant cell formation of Emi1-depleted pluripotent cells can be mimicked by depletion of geminin only, but not by depletion of cyclins A2 or B1. We also compared the DNA content of cells depleted of geminin, cyclin A2, cyclin B1, Emi1, or Oct4 with control siRNA-treated cells by measuring the relative fluorescence intensity of DAPI as a function of nuclear volume in individual nuclei. Interestingly, depletion of Emi1 reproducibly induced the highest accumulation of DNA (Figure 2C). When the mean DNA content of control P19 EC cells was normalized at 2C, Emi1 depletion resulted in a 20-fold mean relative increase in DNA content at 6 days, with a maximum observed of 188C.

Because depletion of geminin or Emi1 induces overreplication of DNA in human somatic cells [22–24], we asked whether overreplication might be the trigger for inducing trophectoderm markers in mouse pluripotent cells. P19 EC cells were cotransfected with siRNA duplexes targeting both Emi1 and the replication protein Cdc6, a component of the prereplication complex that is essential for the initiation of DNA replication [30]. After codepleting Emi1 and Cdc6, both nuclear size and DNA content at 2 days after siRNA transfection were similar to control cells (Figure 2D; Figure S2E). However, blocking DNA replication in Emi1-depleted cells, either by using aphidicolin or by codepleting Emi1 and Cdc6, did not inhibit upregulation of trophectoderm markers (Figure 2D; Figures S2E and S2F). Hence, although acquisition of a high DNA content is impaired in EC cells treated with aphidicolin or codepleted of Emi1 and Cdc6, induction of trophectoderm markers in Emi1-depleted cells is independent of the effects of Emi1 on DNA replication.

**Figure 3:**
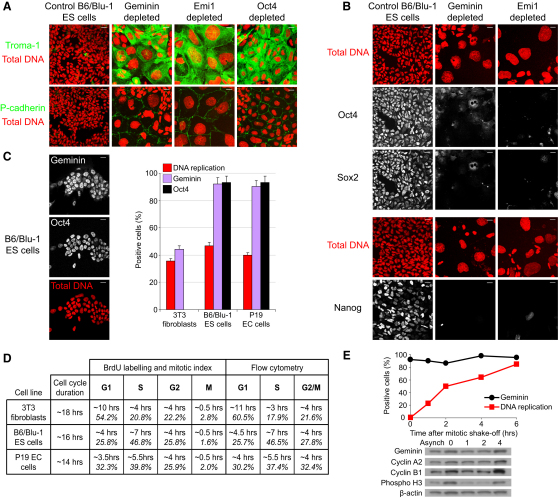
Mouse Embryonic Stem Cells Lose Expression of the Pluripotency Markers Oct4, Sox2, and Nanog and Express Trophoblast Markers on Depletion of Geminin and Emi1 (A) Giant cell formation and induction of trophectoderm markers Troma-1 and P-cadherin (both in green) is evident in B6/Blu-1 mouse embryonic stem (ES) cells depleted of Emi1, geminin, and Oct4 at 2 days. Total DNA is shown in red. Scale bars represent 20 μm. (B) Emi1 and geminin depletion in mouse B6/Blu-1 ES cells result in downregulation of the pluripotency transcription factors Oct4, Sox2, and Nanog (in white). Total DNA is shown in red. Scale bars represent 20 μm. (C) In B6/Blu-1 ES cells, geminin is coexpressed with Oct4 (geminin and Oct4 both in white). Total DNA is shown in red. Scale bars represent 20 μm. The bar chart shows that geminin is present in a higher proportion of asynchronous ES and EC cells than in 3T3 fibroblasts, and notably in a far great proportion of cells that are not actively replicating DNA. Error bars represent 5% standard error. (D) Table summarizing cell-cycle analyses by immunofluorescence and flow cytometry. In asynchronous populations, the majority of 3T3 fibroblasts are in G1, in contrast to ES and EC cells in which G1 comprises a small fraction of the cell cycle and the majority of cells are in S phase. (E) Geminin is present during G1 in ES cells harvested at different time points following mitotic shake-off. Approximately 50% of ES cells enter S phase at 2 hr, suggesting that the duration of G1 phase is likely to be between 2 and 4 hr. For additional related data, see Figure S3.

Following our initial observations in mouse EC cells, we then examined the phenotypic consequences of depleting geminin, Emi1, or Oct4 in B6/Blu-1 mouse ES cells. Depletion of geminin or Emi1 in B6/Blu-1 ES cells also mimics depletion of the pluripotency factor Oct4 in causing upregulation of trophectoderm markers at 2 days (Figure 3A; Figure S3A) and at 6 days (Figure S3B), but not in the extent of nuclear enlargement observed. Blocking DNA replication by codepletion of Cdc6 and by using aphidicolin (data not shown) again demonstrated that this effect on ES cells does not depend on DNA replication, as observed in EC cells. Although Troma-1 and P-cadherin were clearly induced, upregulation of Cdx2 or Eomes and other embryonic differentiation markers was not seen (Figure S3A). This suggests that extraembryonic differentiation is not as clear in ES cells as in EC cells or might bypass the need for these transcription factors. In contrast to our results, a recent report using A2lox mouse ES cells did not identify any changes in DNA ploidy or loss of pluripotency following depletion of geminin [31], which might be consistent with the pleiotropic effects that have been described following geminin depletion in somatic cells [32]. However, geminin and Emi1 depletion both result in downregulation of Oct4, Sox2, and Nanog in both B6/Blu-1 ES cells (Figure 3B) and P19 EC cells (Figure S3C), indicating that destabilization of geminin, either directly or by unopposed APC/C^Cdh1^ activity, also causes loss of pluripotent stem cell identity. Our results support the interpretation that geminin null mouse embryos fail to form an inner cell mass because geminin has an additional and essential role in pluripotency [3, 4], rather than only being necessary for cell-cycle progression and preventing overreplication of DNA during embryonic cell divisions [4]. These findings are also consistent with the observation that geminin restricts multilineage commitment in the early *Xenopus* embryo [33].

In mammalian somatic cells, geminin prevents the reinitiation of DNA replication during S phase, G2, and early mitosis [1, 2, 5, 24], when it is degraded at the metaphase-anaphase transition by APC/C^Cdh1^. It is absent from G1. Yet if geminin is required to maintain the expression of pluripotency factors in ES and EC cells and its depletion induces trophoblast differentiation independently of its effects on DNA replication, why don't pluripotent cells differentiate in G1 when geminin is expected to be absent? We therefore explored the possibility that regulation of geminin during the cell cycle in pluripotent cells might differ from its regulation in other cells, as is consistent with a published blot [9]. Chemical synchronization was not possible because ES or EC cells could not be released from cell-cycle arrest. However, Figure 3C and Figure S3D show that geminin is present by immunofluorescence in the vast majority of asynchronous ES and EC cells, at frequencies above 90% and equivalent to the pluripotency factor Oct4, suggesting that it must also be expressed during G1 in pluripotent cells. Although part of the difference observed from somatic cells could conceivably be accounted for by the higher proportion of pluripotent cells in S phase [7], Figure 3C and Figure S3E show that in contrast to 3T3 fibroblasts, geminin persists outside S phase in most proliferating ES and EC cells. To resolve when geminin is present during the cell cycle, we next estimated the duration of individual cell-cycle phases in exponentially growing 3T3 fibroblasts, ES, and EC cells by using a combined approach of short and long pulses for BrdU labeling, scoring labeled mitoses, and flow cytometric analysis. Consistent with published data [7], we found that ES and EC cells proliferated faster than somatic cells and that G1 was truncated, with almost 50% of the cell cycle dedicated to S phase (Figure 3D). The observation that geminin is present in ∼90% of asynchronously proliferating ES and EC cells therefore indicates that geminin must also be present during G1. Mitotic ES cells were also harvested following shake-off and followed through to S phase. Figure 3E confirms that geminin persists during G1 in ES cells. Because cyclins are APC/C^Cdh1^ substrates that are also not degraded in ES cells during G1, S phase, or G2 (Figure 3E) [8, 9], collectively, these results indicate that APC/C^Cdh1^ activity is suppressed in interphase ES cells, particularly because its inhibitor Emi1 also persists during G1 (Figure S3F) unlike in somatic cells when its presence is restricted to S phase, G2, and early mitosis [34, 35]. Notably, geminin is also detectable by immunoblotting at 0, 15, 30, and 45 min following mitotic shake-off. By immunofluorescence, cytoplasmic geminin was observed at variable intensity in 98.4% of metaphase ES cells (n = 64), 70.9% of anaphase ES cells (n = 55), and 49.0% of telophase ES cells (n = 51), further indicating that activity of the APC/C is reduced. During the early embryonic cell cycles of *Xenopus*, a significant fraction of geminin (∼40%) has also been found to remain present during mitotic exit [36].

**Figure 4:**
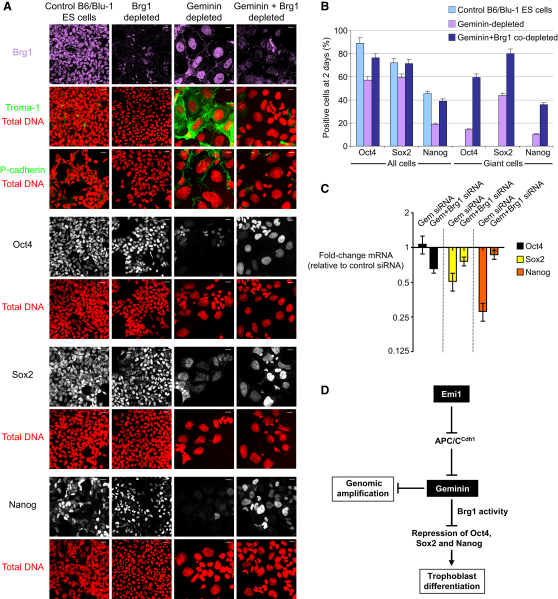
Geminin Antagonizes Brg1 to Inhibit Repression of Oct4, Sox2, and Nanog in B6/Blu-1 ES Cells (A) Codepletion of geminin and Brg1 abolishes the upregulation of trophectoderm markers (in green) observed in geminin-depleted ES cells, but not nuclear enlargement (total DNA, red). Geminin and Brg1 codepletion also blocks the loss of Oct4, Sox2, and Nanog (in white) seen in geminin-depleted ES cells. Scale bars represent 20 μm. (B) Bar chart shows that codepletion of geminin and Brg1 rescues the loss of Oct4, Sox2, and Nanog induced by depletion of geminin alone, particularly in enlarged nuclei. (C) Codepletion of geminin and Brg1 rescues loss of Sox2 and Nanog in geminin-depleted ES cells. Error bars indicate standard error of the mean using four housekeeping genes for normalization. (D) Model of how geminin maintains genomic stability and the pluripotent state in ES cells. Suppressed APC/C^Cdh1^ activity, possibly mediated by Emi1, stabilizes geminin in ES cells to prevent overreplication of DNA and inhibit repression of the core pluripotency factors Oct4, Sox2, and Nanog by antagonizing the chromatin-remodeling protein Brg1. For additional related data, see Figure S4.

Geminin inhibits neural differentiation in P19 EC cells and *Xenopus* embryos by antagonizing the activity of Brg1 (Brahma-related gene 1) [15], the ATPase catalytic subunit of the SWI/SNF chromatin-remodeling complex that associates with sequence-specific transcription factors, histone acetyltransferases, and histone deacetylases to activate or repress the expression of target genes [37]. Interestingly, Brg1 also binds regulatory elements in the *Oct4* promoter to repress Oct4 transcription in the developing trophectoderm of preimplantation mouse embryos [38]. We therefore reasoned that geminin might maintain the undifferentiated state and prevent trophoblast differentiation in ES cells by antagonizing Brg1 and blocking Brg1-mediated repression of Oct4. When we examined Brg1 levels in ES and EC cells depleted of geminin, Brg1 levels did not change (Figure S4A). However, cotransfection of ES cells with siRNA duplexes targeting both geminin and Brg1 abolishes the upregulation of trophectoderm markers (Figures 4A and 4B). Thus, differentiation in response to geminin depletion depends on Brg1. In contrast, the effect of geminin depletion on overreplication of DNA is independent of Brg1. Moreover, Brg1 is required for geminin depletion to downregulate Oct4, Sox2, and Nanog (Figures 4A and 4B), an effect also observed at the transcriptional level for Sox2 and Nanog (Figure 4C). Hence, although Brg1 is not required for overreplication in geminin-depleted cells, downregulation of Oct4, Sox2, and Nanog in geminin-depleted pluripotent cells requires Brg1.

Our findings indicate that geminin plays a pivotal role in a pluripotency pathway that links the cell cycle to expression of core pluripotency factors. We propose a model (Figure 4D) in which suppression of APC/C^Cdh1^ activity during interphase in ES cells, possibly mediated by Emi1, ensures that geminin escapes proteasomal destruction and remains stabilized during G1. This extends the presence of geminin through most of the cell cycle, antagonizing Brg1-mediated chromatin remodeling and preventing repression of Oct4, Sox2, and Nanog.

Our results explain one paradox but reveal another. We show that the requirement for geminin to maintain pluripotency is satisfied by retaining geminin in G1 of pluripotent cells, in contrast to other cell types. However, these results raise a second question of how DNA replication is coupled to the cell cycle in the absence of oscillations of the proteins that normally achieve this, namely geminin and cyclin A2. The extended presence of geminin through the cell cycle also raises further interesting questions, such as when and how prereplication complexes assemble in ES cells. In mammalian somatic cells, APC/C^Cdh1^-mediated destruction of geminin during mitosis allows recruitment of Cdt1 onto chromatin and formation of new prereplication complexes [1, 2]. We therefore speculate that geminin might only be absent in mouse ES cells for a very short window during late telophase, or in very early G1, to allow prereplication complexes to assemble. Alternatively, posttranslational modifications similar to those observed for *Xenopus* geminin, which is not destroyed in mitotic egg extracts but inactivated by ubiquitination [39], might prevent the binding of geminin to Cdt1 during G1 in mammalian ES cells without affecting its ability to antagonize Brg1. A further question emerges: How is suppression of APC/C activity compatible with progression through the cell cycle in ES cells when oscillations in each of its substrates are lost? Such new questions into the control of the cell cycle in ES cells demand urgent attention, particularly when considering novel approaches for enhancing the efficiency of nuclear reprogramming by defined transcription factors in the generation of iPS cells.

## Experimental Procedures

### Cell Culture

P19 EC cells (American Type Culture Collection) were grown in α-modified Eagle's medium (Sigma-Aldrich) supplemented with 7.5% donor bovine serum, 2.5% fetal bovine serum (FBS), 2 mM L-glutamine, 100 units/ml penicillin, and 0.1 mg/ml streptomycin (GIBCO). NIH 3T3 fibroblasts (European Collection of Cell Cultures) were grown in Dulbecco's modified Eagle's medium (DMEM; GIBCO) supplemented with 15% fetal calf serum, 100 units/ml penicillin, and 0.1 mg/ml streptomycin. B6/Blu-1 ES cells were maintained in KnockOut DMEM (GIBCO), supplemented with 15% FBS, 10 pM β-mercaptoethanol (Sigma-Aldrich), 1000 U/ml LIF (Millipore), 2 mM L-glutamine (AMRESCO), 0.012 g/ml penicillin, and 0.02 g/ml streptomycin on 0.1% gelatin-coated flasks.

### RNA Interference

siRNA duplexes were synthesized by QIAGEN and designed to target the relevant sequences (see Supplemental Experimental Procedures). Control siRNAs were designed by substitution of two base pairs for each sequence. P19 EC cells, NIH 3T3 fibroblasts, and B6/Blu-1 ES were transfected using Oligofectamine (Invitrogen), Lipofectamine RNAiMax (Invitrogen), or “Nucleofector” technology (Lonza) according to manufacturers' instructions.

### Immunoblotting and Immunofluorescence

For immunoblotting, cells were lysed directly in 2× Laemmli sample buffer (350 nM Tris-HCl [pH 6.5], 10% glycerol, 3% sodium dodecyl sulfate [SDS], 3% dithiothreitol, 2.7% bromophenol blue), boiled, and run on polyacrylamide Tris-glycine gels (Invitrogen). Proteins were transferred to a polyvinylidene difluoride membrane. Blocking of membranes and incubation steps with the appropriate primary antibodies (see Supplemental Experimental Procedures) and HRP-conjugated secondary antibodies (Dako) were performed in 5% milk in 0.1% Tween 20/phosphate-buffered saline. Antibodies were detected using ECL (GE Healthcare).

For BrdU or EdU immunofluorescence, cells were incubated with 20 μM BrdU or 10 μM EdU prior to fixation in 4% paraformaldehyde. For immunofluorescent staining of cyclin E, cells were permeabilized with 0.1% Triton X-100 on ice prior to fixation. Cells were permeabilized with 0.1% Triton X-100 and 0.02% SDS, blocked with 1% BSA (Sigma-Aldrich), and incubated with primary antibody. Coverslips were incubated with Alexa Fluor-conjugated secondary antibodies (Molecular Probes) and DAPI (Sigma-Aldrich), mounted with fluorescence mounting medium (Dako), and sealed with nail varnish. Confocal images were collected on a Zeiss LSM 510 META confocal microscope and rendered using its image browser software (Carl Zeiss MicroImaging). Serial confocal sections (Z stacks) were collected at 0.25 μm intervals and compiled into a single image for semiquantitative assessment of DNA content using Volocity LE 3.0.2 software (Improvision).

### Cell-Cycle Analysis

The average length of a single cell cycle was calculated as the time required for exponentially proliferating cells to double in number. The S phase fraction was determined by the proportion of asynchronously proliferating cells that had incorporated BrdU after a 20 min pulse. Mitotic cells were quantified by DAPI staining. Fractions were then multiplied by the total length of the cell cycle to obtain estimates of the duration of each phase. The average length of G2 was estimated from the length of BrdU pulse required to label half the mitotic cells in asynchronously proliferating cell populations [40]. G1 was then calculated by subtracting the lengths of S phase, G2, and mitosis from the length of a single cell cycle. For flow cytometry, cells were trypsinized and fixed in ice-cold 70% methanol before staining with propidium iodide (Sigma-Aldrich) and analyzed for DNA content using BD LSR II and FACSDiva software (Becton Dickinson).

## Supplemental Information

Document S1. Four Figures and Supplemental Experimental Procedures
