# Supplementary material for: Geminin Escapes Degradation in G1 of Mouse Pluripotent Cells and Mediates the Expression of Oct4, Sox2, and Nanog
Source: Curr Biol. 2011 Apr 26;21(8):692–9. doi: 10.1016/j.cub.2011.03.026 (PMC3083515; doi:10.1016/j.cub.2011.03.026)
Supplement: Document S1. Four Figures and Supplemental Experimental Procedures [file mmc1.pdf]

Current Biology, Volume *21*

Supplemental Information

Geminin Escapes Degradation in G1 of Mouse Pluripotent Cells and Mediates the Expression of Oct4, Sox2, and Nanog

Valerie S. Yang, Stephanie A. Carter, Sarah J. Hyland, Kikuë Tachibana-Konwalski, Ronald A. Laskey, and Michael A. Gonzalez

Figure S1

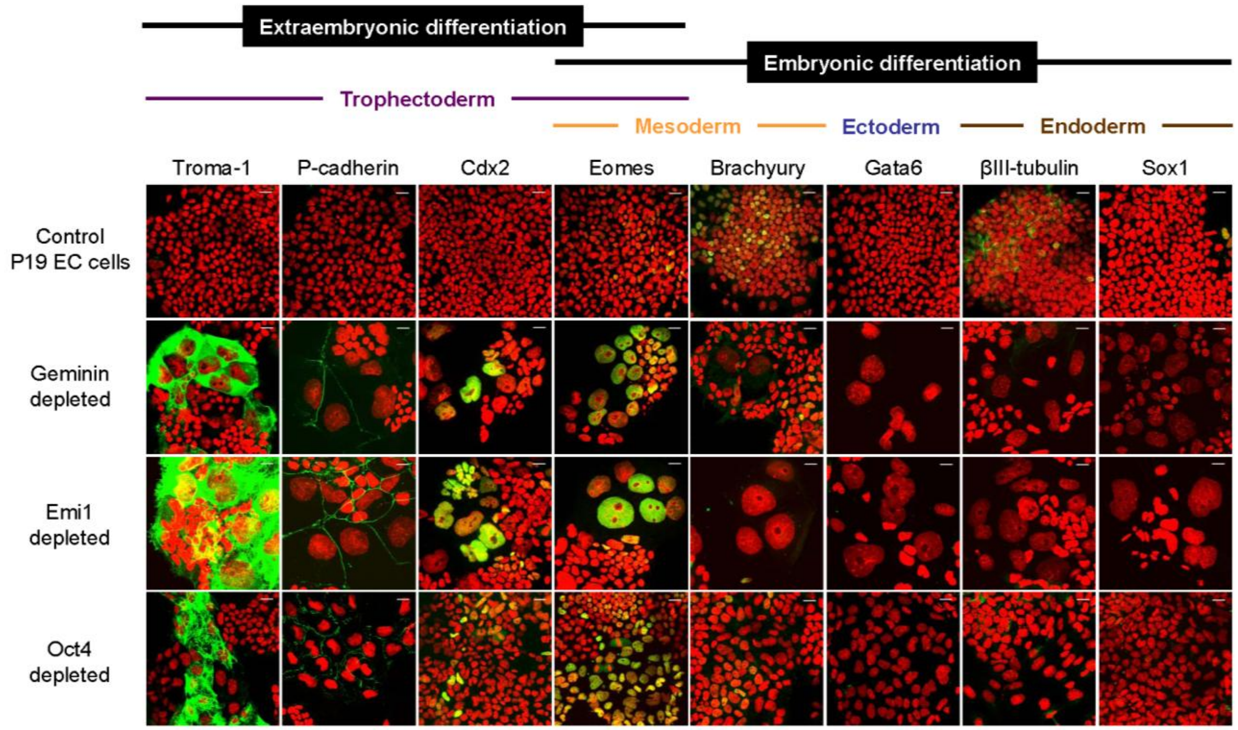

Figure S1, Related to Figure 1. Lineage Analysis of P19 EC Cells Depleted of Geminin, Emi1, and Oct4

Depletion of geminin, Emi1, and Oct4 at 2 days induces upregulation of trophoblast markers Troma-1, P-cadherin, Cdx2, and Eomes but not markers indicative of differentiation towards embryonic lineages. Total DNA is shown in red. Scale bar represents 20 μm.

Figure S2

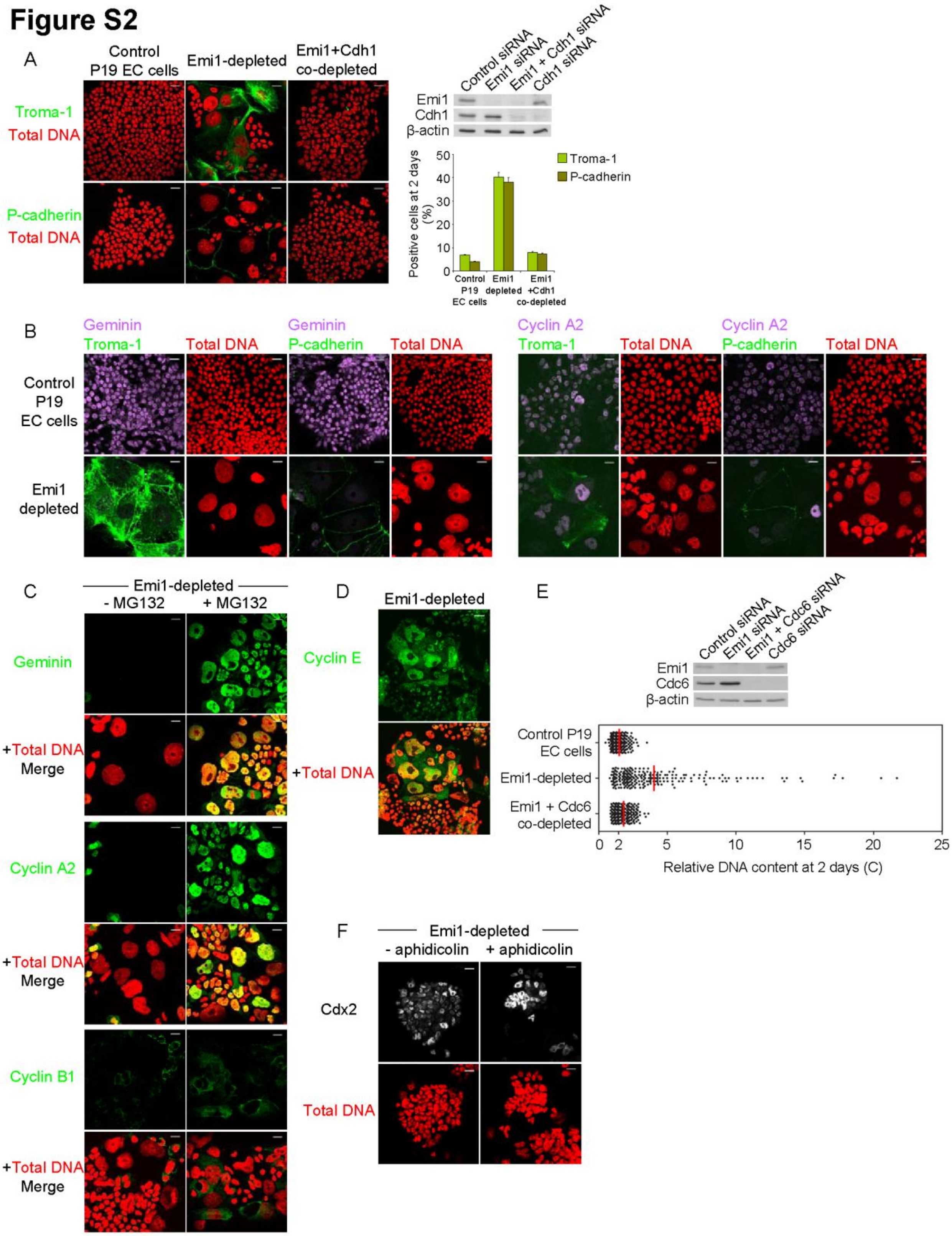
A
Control P19 EC cells
Emi1-depleted
Emi1+Cdh1 co-depleted
Troma-1
Total DNA
P-cadherin
Total DNA
Control siRNA
Emi1 siRNA
Emi1 + Cdh1 siRNA
Cdh1 siRNA
Emi1
Cdh1
β-actin
Positive cells at 2 days (%)
Troma-1
P-cadherin
Control P19 EC cells
Emi1 depleted
Emi1 +Cdh1 co-depleted
B
Geminin
Troma-1
Total DNA
Geminin
P-cadherin
Total DNA
Cyclin A2
Troma-1
Total DNA
Cyclin A2
P-cadherin
Total DNA
Control P19 EC cells
Emi1 depleted
C
Emi1-depleted
- MG132
+ MG132
Geminin
+Total DNA Merge
Cyclin A2
+Total DNA Merge
Cyclin B1
+Total DNA Merge
D
Emi1-depleted
Cyclin E
+Total DNA
E
Control siRNA
Emi1 siRNA
Emi1 + Cdc6 siRNA
Cdc6 siRNA
Emi1
Cdc6
β-actin
Control P19 EC cells
Emi1-depleted
Emi1 + Cdc6 co-depleted
Relative DNA content at 2 days (C)
F
Emi1-depleted
- aphidicolin
+ aphidicolin
Cdx2
Total DNA

Figure S2, Related to Figure 2. Emi1 Depletion Results in Nuclear Enlargement, Upregulation of Trophectoderm Markers, and Unopposed APC/C^{Cdh1} Activity in Mouse EC Cells

(A) Emi1, Cdh1 and both were depleted by siRNA in P19 EC cells. Codepleting Emi1 and Cdh1 antagonizes the effect of depleting Emi1 alone in P19 EC cells, inhibiting both nuclear enlargement (total DNA is shown in red) and induction of trophectoderm markers Troma-1 and P-cadherin (both in green) at two days posttransfection. Scale bar represents 20 μm. Whole cell lysates were harvested for analysis by western blotting with Emi1 and Cdh1 antibodies. β-actin was used as a loading control. Bar chart shows the proportion of control, Emi1-depleted, and Emi1 and Cdh1 codepleted P19 EC cells labelled with Troma-1 and P-cadherin by immunofluorescence. At least 1000 cells were scored for each time point. Error bars represent 5% standard error.

(B) Emi1 depletion results in massive nuclear enlargement (total DNA is shown in red) and upregulation of trophectoderm markers, Troma-1 and P-cadherin (in green) specifically in cells in which the APC/C^{Cdh1} substrates geminin or cyclin A2 (in purple) are downregulated. Scale bar represents 20 μm.

(C) Geminin, cyclin A2, and cyclin B1 (in green) are downregulated following depletion of Emi1 in P19 EC cells and dramatically stabilized following MG132 treatment. Total DNA is shown in red. Scale bar represents 20 μm.

(D) Cyclin E (green), which is not a target of the APC/C, is upregulated by immunofluorescence in enlarged nuclei (total DNA is shown in red) following depletion of Emi1 in pluripotent cells. Scale bar represents 20 μm.

(E) Emi1, Cdc6 and both were depleted by siRNA in P19 EC cells and whole cell lysates were harvested for analysis by western blotting. β-actin was used as a loading control. Scatter dot plots show relative DNA content analysis of control, Emi1-depleted, and Emi1 and Cdc6 codepleted P19 EC cells. Z stacks of DAPI-stained nuclei were collected and compiled into a single 3D-reconstructed image for analysis. Red bars represent the mean value.

(F) Cdx2 (in white) is induced following depletion of Emi1 at 24 hours posttransfection and remains upregulated in Emi1-depleted cells treated with aphidicolin for 12 hours at 12 hours posttransfection. Total DNA is shown in red. Scale bar represents 20 μm.

Figure S3

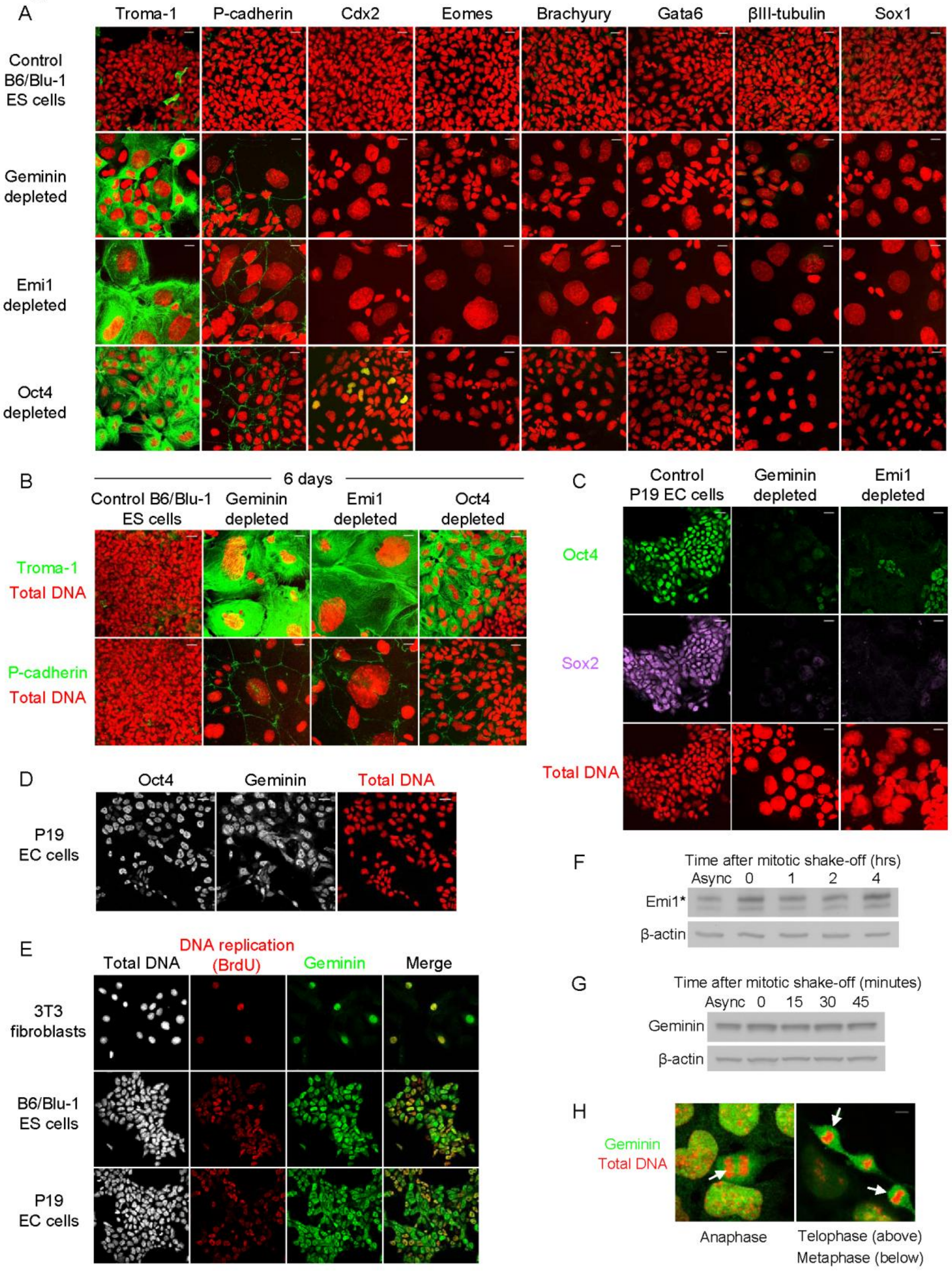
A
Troma-1
P-cadherin
Cdx2
Eomes
Brachyury
Gata6
βIII-tubulin
Sox1
Control B6/Blu-1 ES cells
Geminin depleted
Emi1 depleted
Oct4 depleted
B
6 days
Control B6/Blu-1 ES cells
Geminin depleted
Emi1 depleted
Oct4 depleted
Troma-1
Total DNA
P-cadherin
Total DNA
C
Control P19 EC cells
Geminin depleted
Emi1 depleted
Oct4
Sox2
Total DNA
D
Oct4
Geminin
Total DNA
P19 EC cells
E
Total DNA
DNA replication (BrdU)
Geminin
Merge
3T3 fibroblasts
B6/Blu-1 ES cells
P19 EC cells
F
Time after mitotic shake-off (hrs)
Async 0 1 2 4
Emi1*
β-actin
G
Time after mitotic shake-off (minutes)
Async 0 15 30 45
Geminin
β-actin
H
Geminin
Total DNA
Anaphase
Telophase (above)
Metaphase (below)

Figure S3, Related to Figure 3. Mouse Pluripotent Cells Lose Expression of Pluripotency Markers and Express Trophoblast Markers on Depletion of Geminin and Emi1

(A) Lineage analysis of B6/Blu-1 ES cells depleted of geminin, Emi1, and Oct4. Total DNA is shown in red. Scale bar represents 20 μm.

(B) Trophectoderm markers Troma-1 and P-cadherin (in green) are present at 6 days in B6/Blu-1 ES cells depleted of geminin, Emi1, and Oct4. Total DNA is shown in red. Scale bar represents 20 μm.

(C) Loss of Oct4 and Sox2 is observed following depletion of geminin or Emi1 in P19 EC cells. Nanog was not found to be present in control P19 EC cells.

(D) Geminin and Oct4 (in white) are coexpressed in proliferating P19 EC cells. Total DNA is shown in red. Scale bar represents 20 μm.

(E) Geminin (in green) is present in a higher proportion of asynchronous ES and EC cells than in 3T3 fibroblasts and notably in a much greater proportion of cells that are not actively replicating DNA. Total DNA is shown in red. Scale bar represents 20 μm.

(F) Emi1 is present during G1 in ES cells harvested at different time points following mitotic shake-off. A cross-reacting band lies below the Emi1 band marked *.

(G) Geminin is present by immunoblotting at 0, 15, 30, and 45 minutes in ES cells harvested following mitotic shake-off.

(H) Geminin (in green) is present following the metaphase:anaphase transition in B6/Blu-1 ES cells. Total DNA is shown in red. Scale bar represents 20 μm.

Figure S4

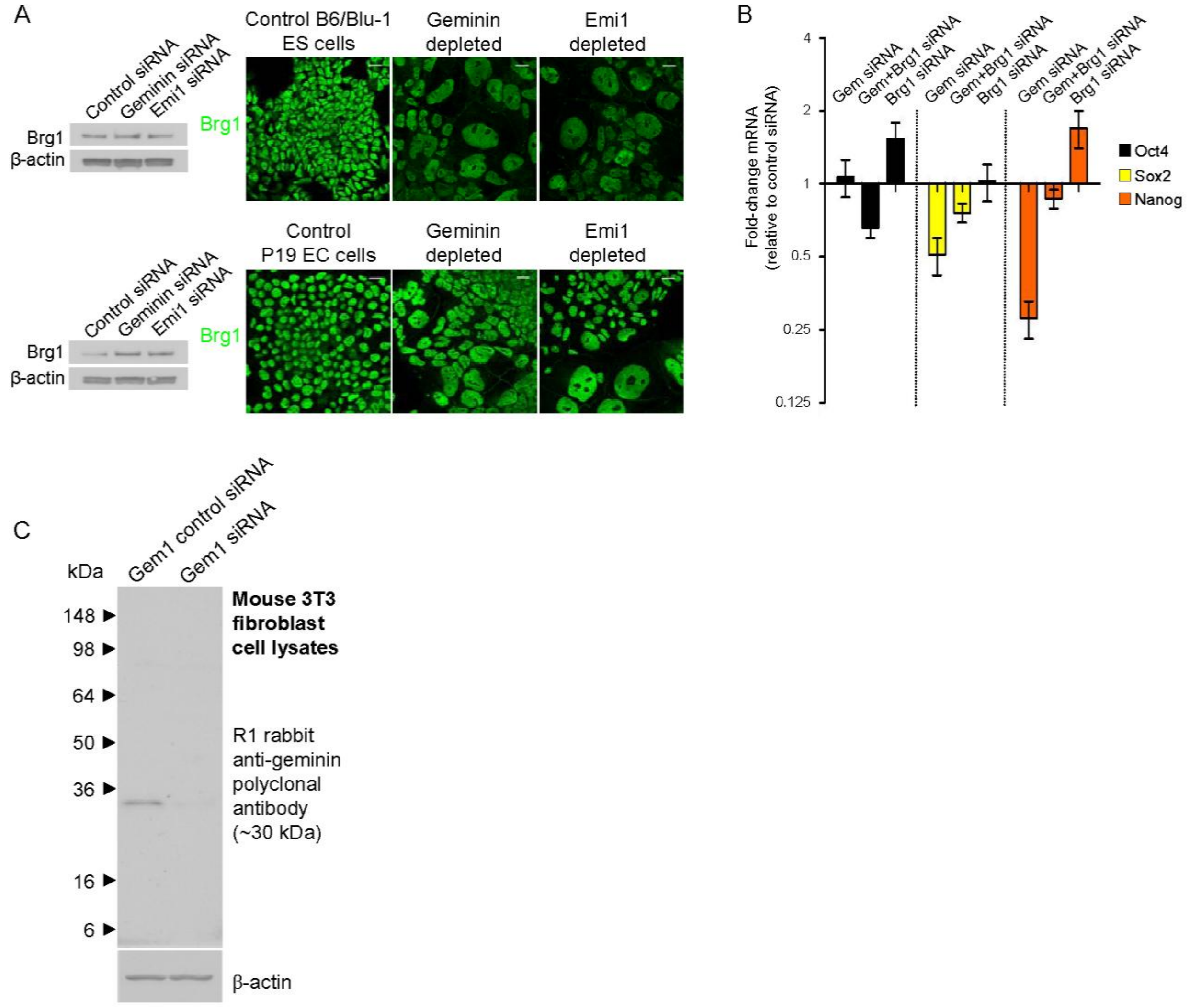

Figure S4, Related to Figure 4. Geminin Antagonizes Brg1 to Inhibit Repression of Oct4, Sox2, and Nanog in B6/Blu-1 ES Cells

(A) Geminin and Emi1 were depleted by siRNA in P19 EC and B6/Blu-1 ES cells. Whole cell lysates were harvested for analysis by western blotting with Brg1 antibody. β-actin was used as a loading control.

(B) Codepletion of geminin and Brg1 rescues loss of Sox 2 and Nanog in geminin-depleted ES cells. Error bars indicate standard error of the mean using four housekeeping genes for normalization.

(C) Validation of rabbit polyclonal anti-geminin antibody by immunoblotting. Western blots of whole cell lysates from mouse 3T3 cells transfected with control or geminin siRNA were incubated with immunoaffinity-purified R1 anti-mouse geminin antibody. β-actin was used as a loading control.

Supplemental Experimental Procedures

RNA Interference

siRNA duplexes were designed to target the following sequences:

Emi1: TACAAAGATTGTGATAGATCA, ACCGTGGACGGTTGTAAAGAA, CAGCGGCATGGACTTAGTAAA and GTGGGACATGATAACAAGGAA.

Geminin: CAGGAATTTGATTCTGAAGAA, CAGGAAGCCTTTGATCTTATA, CCGCCTGAGAAAGGAGAATAA and ATCGAGAGGCTGAGTAATGAA.

Cyclin A2: CTGGACGGGTTGCTCCTCTTA, TTCGAAGTTTGAAGAAATATA, TTGATTGATTATTCTAAGCAA and CAGACGATACCTATTCCAAGA.

Cyclin B1: CTCACTCTAGTTTAAACTCTA, TACATCTGATATCAAGTTGAA, ATGGGTGTGGCTGCCACCATA and GTGTTCTTAAATGATGTTTAA.

Oct4: CCCGGAAGAGAAAGCGAACTA, AACGAGAAGAGTATGAGGCTA and AACCTTCAGGAGATATGCAAA.

All other siRNAs were obtained from the Qiagen HP genome-wide siRNA databank (http://www.qiagen.com).

Antibodies

Full-length mouse geminin cDNA was amplified by PCR from IMAGE clone 3988065 (MRC UK HGMP Resource Centre, Cambridge, UK). Protein expressed in *Escherichia coli* was used for rabbit immunizations (Scottish National Blood Transfusion Service, Penicuik, UK). R1 rabbit polyclonal antibody underwent immunoaffinity purification and its specificity was confirmed by western blotting (Figure S4C). Staining was abrogated by using geminin siRNA in NIH 3T3 cells and by preincubation of antibody with purified recombinant geminin protein (data not shown). Other antibodies used include rabbit anti-Emi1 antibody (Zymed Laboratories, Paisley, UK), mouse anti-β-actin (Abcam, Cambridge, UK), mouse anti-Oct4 (BD Biosciences, Erembodegem, Belgium), rabbit anti-cyclin A2 antibody (a kind gift from Dr. Mark Carrington, Department of Biochemistry, Cambridge UK), mouse anti-Cdx2 (Biogenex, San Ramon, CA), mouse anti-cyclin B1 (GNS1, Santa Cruz Biotechnology, Heidelberg, Germany), mouse anti-cyclin E (E-4, Santa Cruz Biotechnology), rabbit anti-cyclin E (M-20, Santa Cruz Biotechnology), rabbit anti-Skp2 (H-435, Santa Cruz Biotechnology), mouse anti-Cdh1 (Abcam), rabbit anti-Cdc20 (Abcam), mouse anti-P-cadherin (LabVision, Runcorn, UK), goat anti-Sox2 antibody (R&D Systems, Abingdon, United Kingdom), rat anti-Nanog antibody (eBioscience, Hatfield, United Kingdom) and rabbit anti-Brg1 antibody (Millipore, Watford, United Kingdom). Anti-Troma-1 antibody developed by Rolf Kemler was obtained from the Developmental Studies Hybridoma Bank, University of Iowa (Department of Biological Sciences, Iowa City, IA).

Quantitative Reverse Transcriptase (RT)-PCR

Total RNA was extracted using TRIzol reagent (Invitrogen), RNA concentrations and quality determined using the Nanodrop UV Spectrophotometer (Nanodrop Technologies) and cDNA synthesized using the Quantitect Reverse Transcription kit (Qiagen). Quantitative RT-PCR was performed using SYBR® Green JumpStart™ *Taq* ReadyMix™ (Sigma-Aldrich) and reactions were run on an Opticon™ continuous fluorescence detector PCR machine (MJ Research).

Expression ratios of the genes of interest were calculated using the comparative Ct method described by Pfaffl [1], with normalization to the housekeeping genes *GAPDH*, *HMBS*, *HPRT1* and *ACTB* using the following primers:

Geminin: AGCTTTGGGATGACCAGCTA and GCTCTGCCACTTCTTTCCAA.

Oct4: CCAATCAGCTTGGGCTAGAG and CTGGGAAAGGTGTCCCTGTA.

Nanog: ATGCCTGCAGTTTTTCATCC and GAGGCAGGTCTTCAGAGGAA.

Sox2: ACTTTTGTCCGAGACCGAGA and CTCCGGGAAGCGTGTACTTA.

Emi1: CAGTCAGCGTGGTCAGAGAG and GCAGGGAAATTACAGCGAAC.

Brg1: CACCTACCATGCCAACACTG and CGCTTGTCCTTCTTCTGGTC.

GAPDH: AACTTTGGCATTGTGGAAGG and GGATGCAGGGATGATGTTCT.

HMBS: AAGTGGACCTGGTCGTTCAC and GCAAGGTTTCCAGGGTCTTT.

HPRT1: CTTTGCTGACCTGCTGGATT and TATGTCCCCCGTTGACTGAT.

ACTB: AGTGTGACGTTGACATCCGTA and GCCAGAGCAGTAATCTCCTTCT.

Supplemental Reference

1. Pfaffl, M.W. (2001). A new mathematical model for relative quantification in real-time RT-PCR. Nucleic Acids Res *29*, e45.
